# Supplementary material for: Highly efficient anion exchange membrane water electrolyzers via chromium-doped amorphous electrocatalysts
Source: Nat Commun. 2024 Apr 22;15:3416. doi: 10.1038/s41467-024-47736-0 (PMC11035637; doi:10.1038/s41467-024-47736-0)
Supplement: Supplementary file 1 — Supplementary Information [file 41467_2024_47736_MOESM1_ESM.pdf]

Supplementary Information for

Highly efficient anion exchange membrane water electrolyzers via chromium-doped amorphous electrocatalysts

Sicheng Li[1,+], Tong Liu[1,+], Wei Zhang[1,*], Mingzhen Wang[2], Huijuan Zhang[1], Chunlan Qin[1], Lingling Zhang[1], Yudan Chen[1], Shuaiwei Jiang[1], Dong Liu[1], Xiaokang Liu[1], Huijuan Wang[3], Qiquan Luo[4], Tao Ding[1,*] and Tao Yao[1,*]

[1]School of Nuclear Science and Technology, Key Laboratory of Precision and Intelligent Chemistry, National Synchrotron Radiation Laboratory, Hefei National Research Center for Physical Sciences at the Microscale, University of Science and Technology of China. Hefei, P.R. China.

[2]Zhongke Enthalpy (Anhui) New Energy Technology Co., Ltd. Hefei, P.R. China

[3]Experimental Center of Engineering and Materials Science, University of Science and Technology of China. Hefei, P.R. China

[4]Institutes of Physical Science and Information Technology, Anhui University. Hefei, P.R. China

[+]These authors contributed equally: Sicheng Li, Tong Liu.

*Corresponding author. E-mail: zhangw94@ustc.edu.cn; dingtao@ustc.edu.cn; yaot@ustc.edu.cn

Contents

1. Supplementary Figs. S1 to S31

2. Supplementary Tables S1 to S4

3. Supplementary References

Supplementary Figures

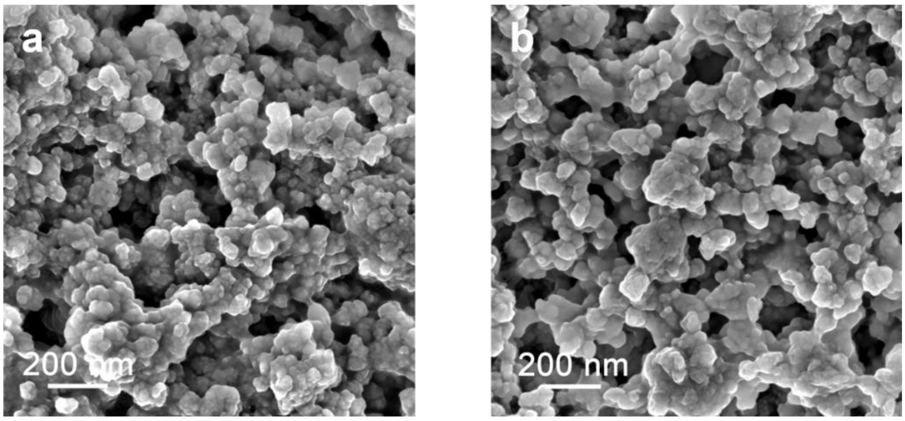

Supplementary Fig. 1. SEM images. SEM images of (a) $FeCrO_x$ and (b) $NiCrO_x$.

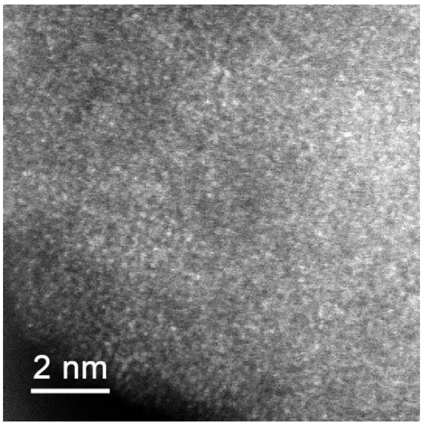

Supplementary Fig. 2. AC HAADF-STEM image. AC HAADF-STEM image of $CoCrO_x$.

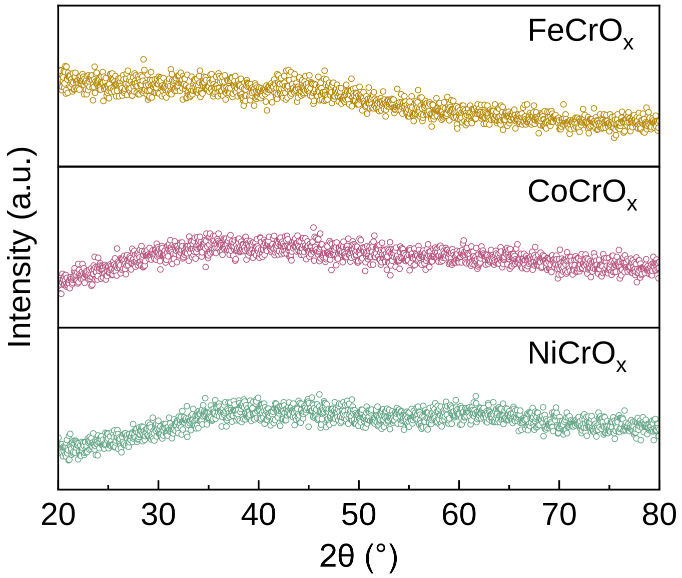

Supplementary Fig. 3. XRD patterns. XRD patterns of $FeCrO_x$, $CoCrO_x$ and $NiCrO_x$.

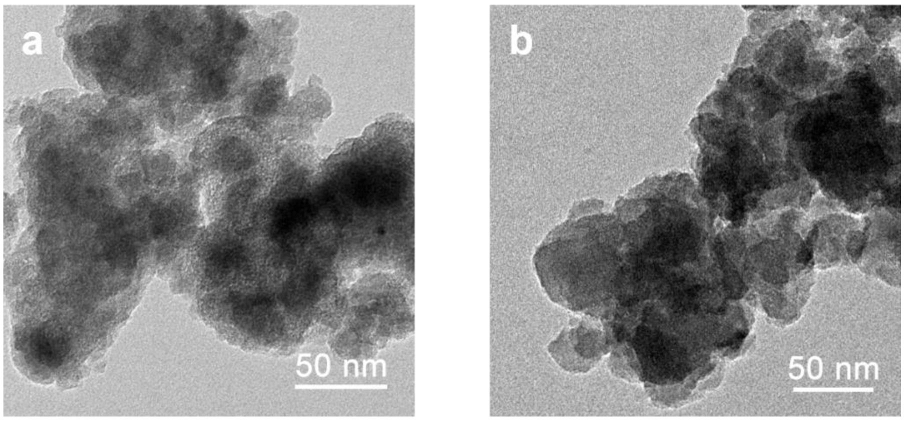

Supplementary Fig. 4. TEM images. TEM images of (a) $FeCrO_x$ and (b) $NiCrO_x$.

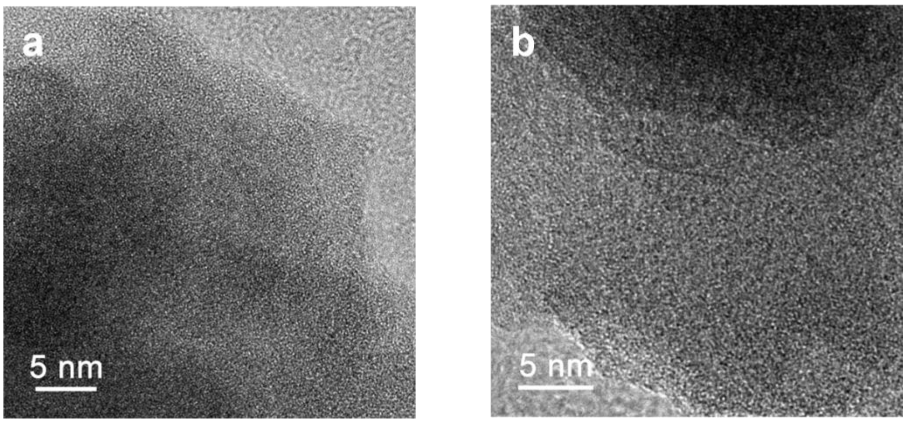

Supplementary Fig. 5. HRTEM images. HRTEM images of (a) $FeCrO_x$ and (b) $NiCrO_x$.

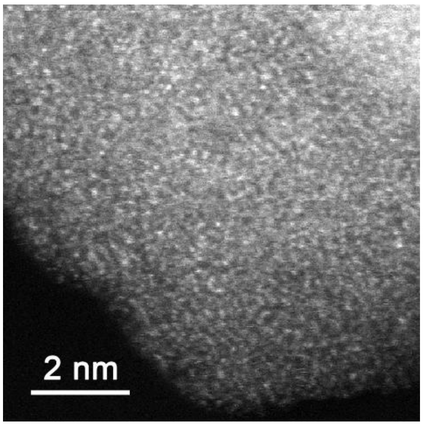

Supplementary Fig. 6. AC HAADF-STEM image. AC HAADF-STEM image of $NiCrO_x$.

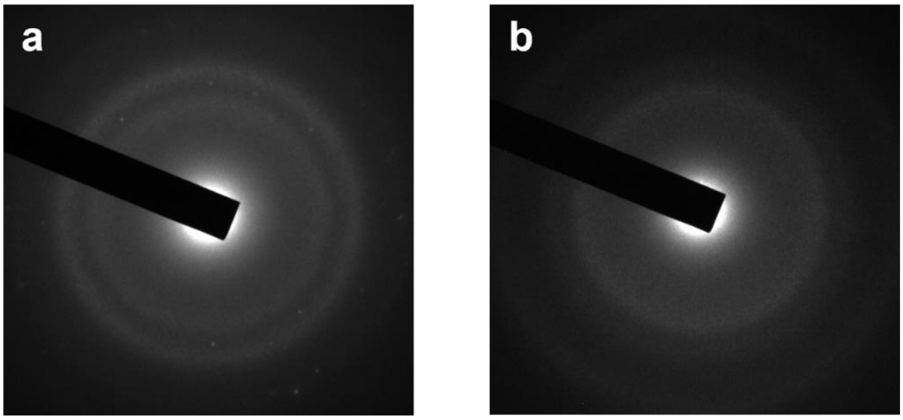

Supplementary Fig. 7. SAED patterns. SAED patterns of (a) $FeCrO_x$ and (b) $NiCrO_x$.

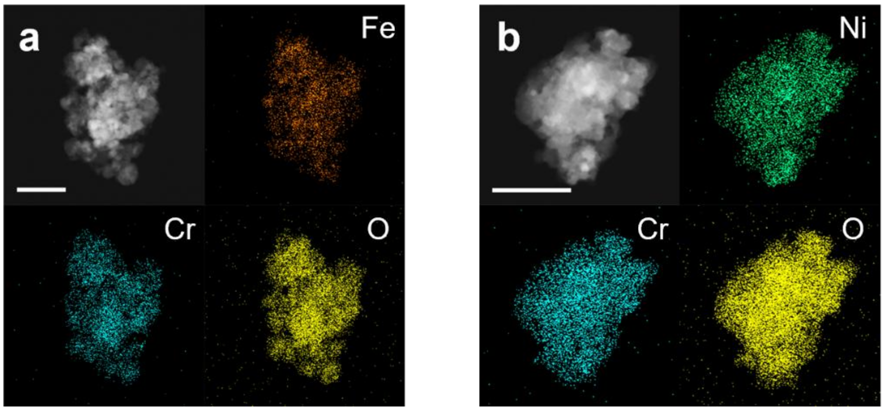

Supplementary Fig. 8. HRTEM images and corresponding EDS mappings. HRTEM images and corresponding EDS mappings of (a) $FeCrO_x$ and (b) $NiCrO_x$ showing the uniform dispersion of Fe (orange) or Ni (green), Cr (blue-green) and O (yellow), respectively. Scale bar: 100 nm.

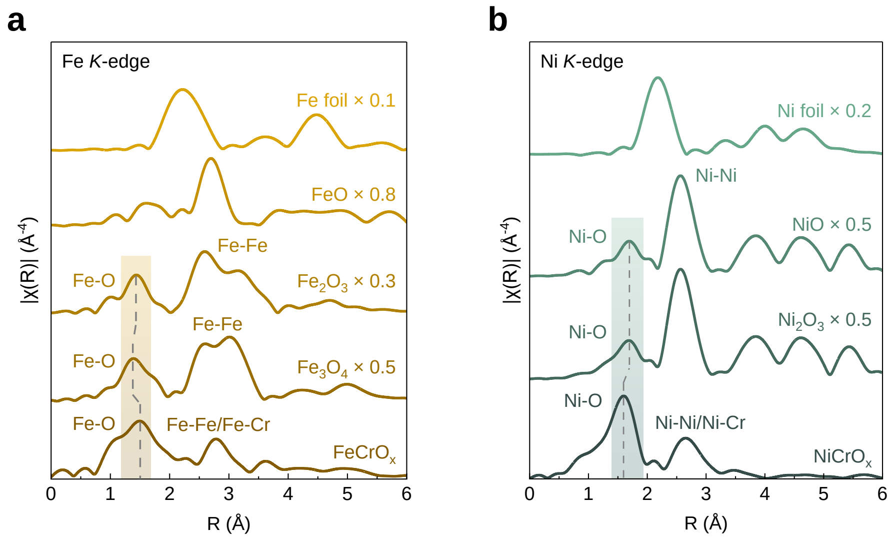

Supplementary Fig. 9. FT-EXAFS spectra. FT-EXAFS spectra of (a) Fe *K*-edge for $FeCrO_x$ and referenced samples and (b) Ni *K*-edge for $NiCrO_x$ and referenced samples.

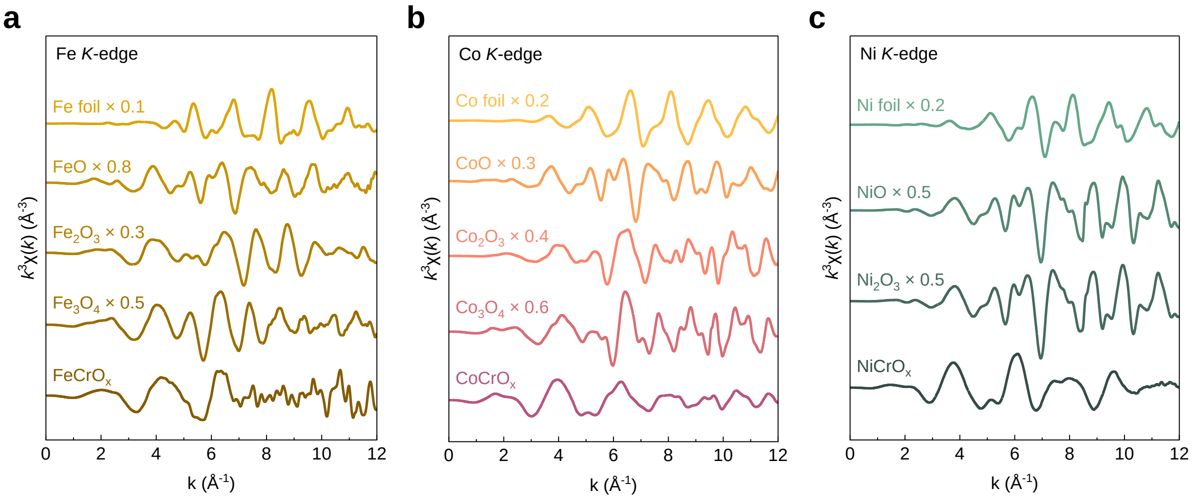

Supplementary Fig. 10. $k^3\chi(k)$ oscillations. $k^3\chi(k)$ oscillations of (a) Fe *K*-edge for $FeCrO_x$ and referenced samples, (b) Co *K*-edge for $CoCrO_x$ and referenced samples and (c) Ni *K*-edge for $NiCrO_x$ and referenced samples.

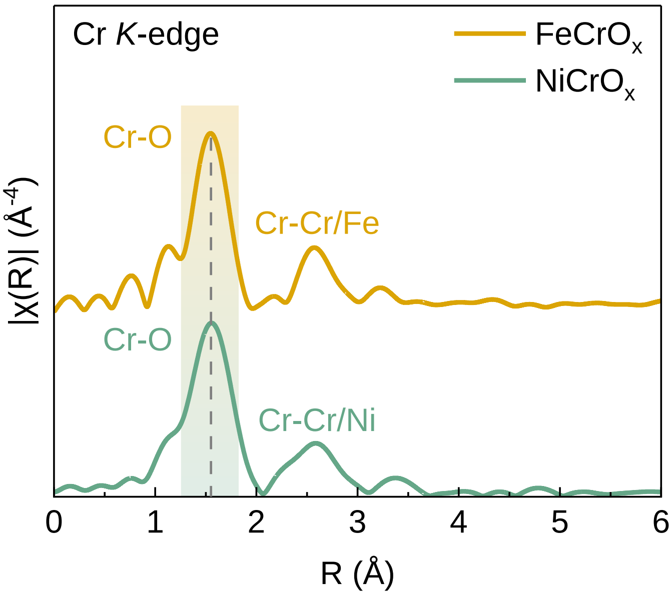

Supplementary Fig. 11. FT-EXAFS spectra. FT-EXAFS spectra of Cr *K*-edge for $FeCrO_x$ and $NiCrO_x$.

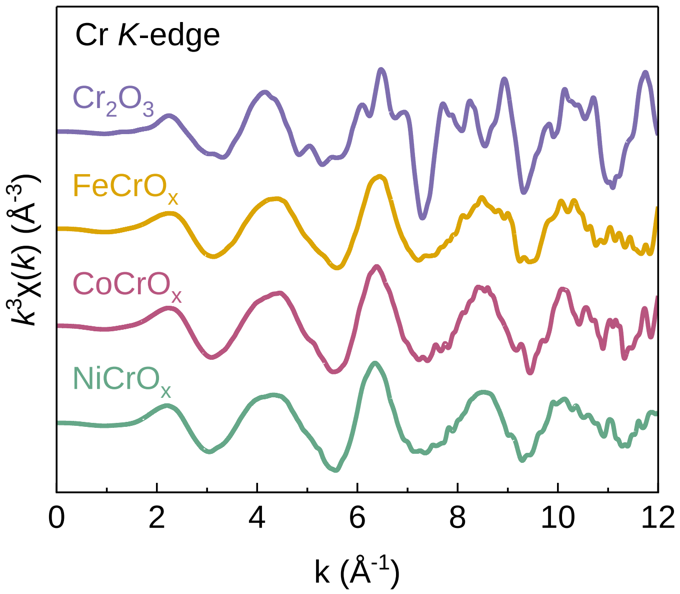

Supplementary Fig. 12. $k^3\chi(k)$ oscillations. $k^3\chi(k)$ oscillations of Cr *K*-edge for $FeCrO_x$, $CoCrO_x$, $NiCrO_x$ and Cr_2O_3 reference sample.

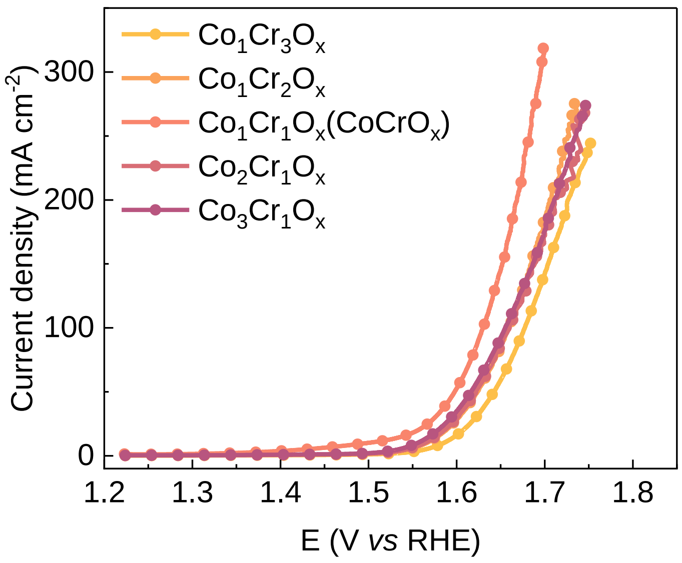

Supplementary Fig. 13. LSV curves. LSV curves for catalysts with different Co and Cr ratios (Co: Cr=1:3~3:1).

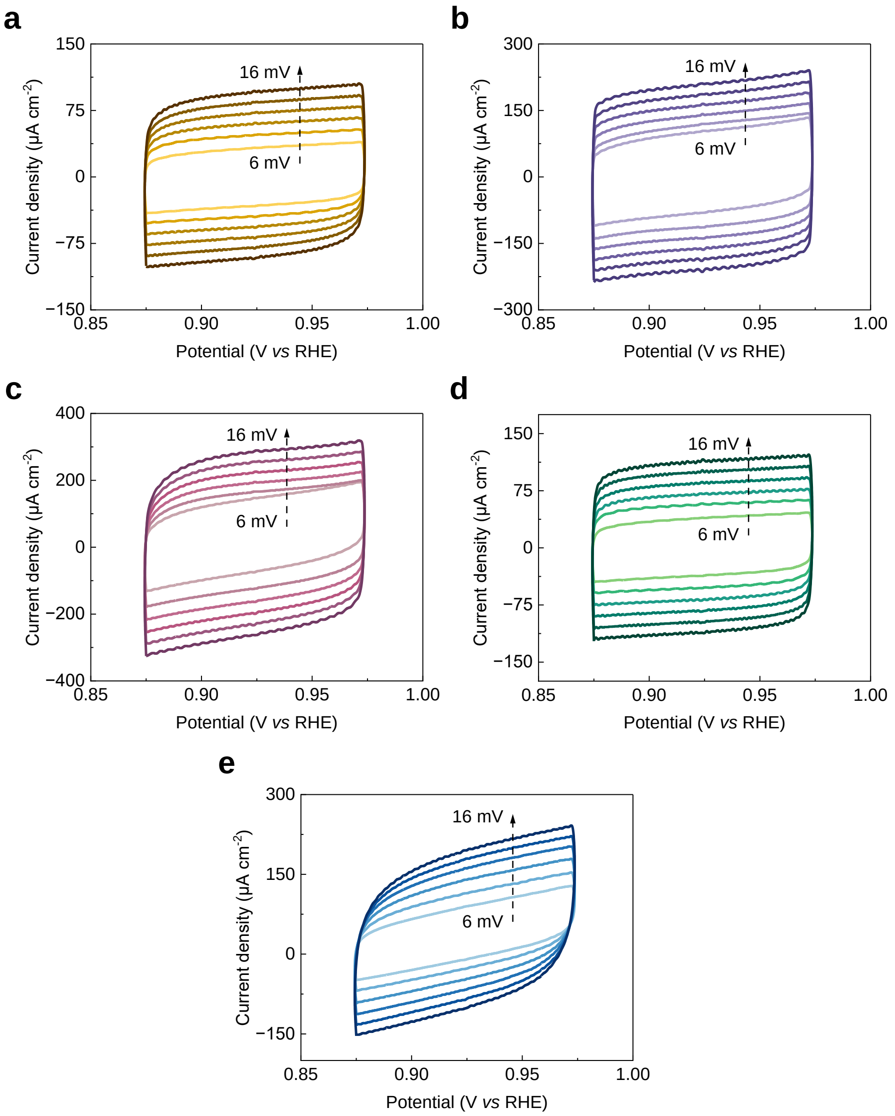

Supplementary Fig. 14. CV curves. CV curves of (a) $FeCrO_x$, (b) CoO_x, (c) $CoCrO_x$, (d) $NiCrO_x$ and (e) RuO_2 acquired in the potential range from 0.875 to 0.975 V (*vs* RHE).

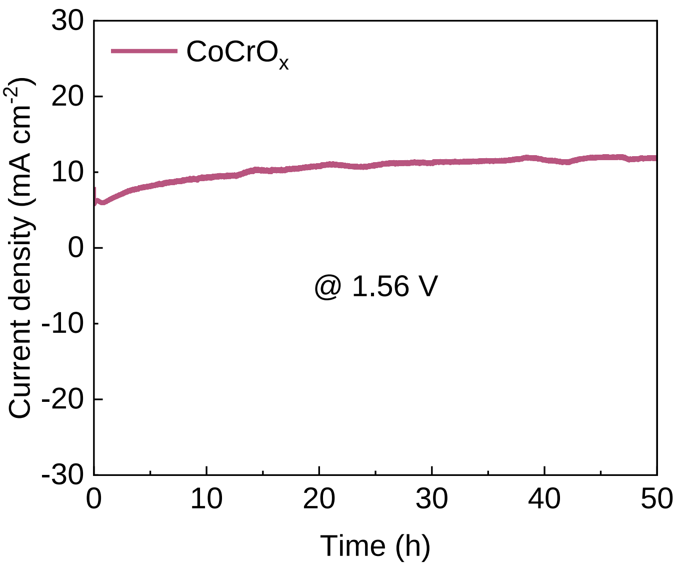

Supplementary Fig. 15. Chronoamperometry curve. Chronoamperometry curve of $CoCrO_x$ at 1.56 V (*vs* RHE).

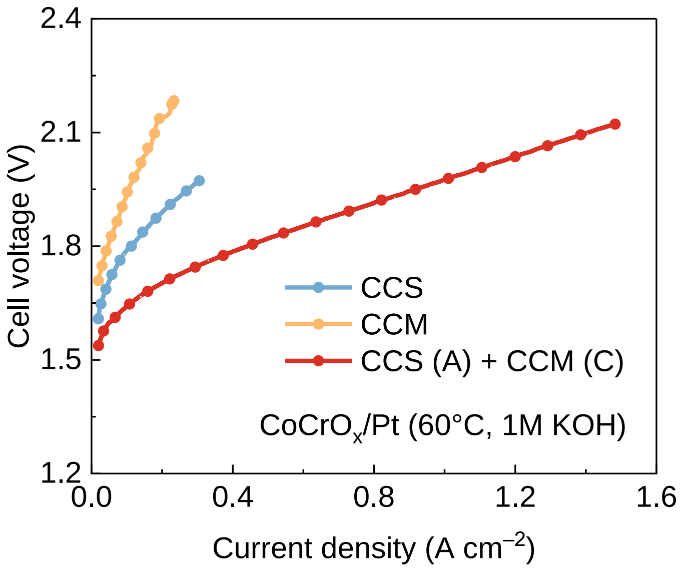

Supplementary Fig. 16. I-V curves. I-V curves of AEMWEs assembled using various methods with $CoCrO_x$ as the anode.

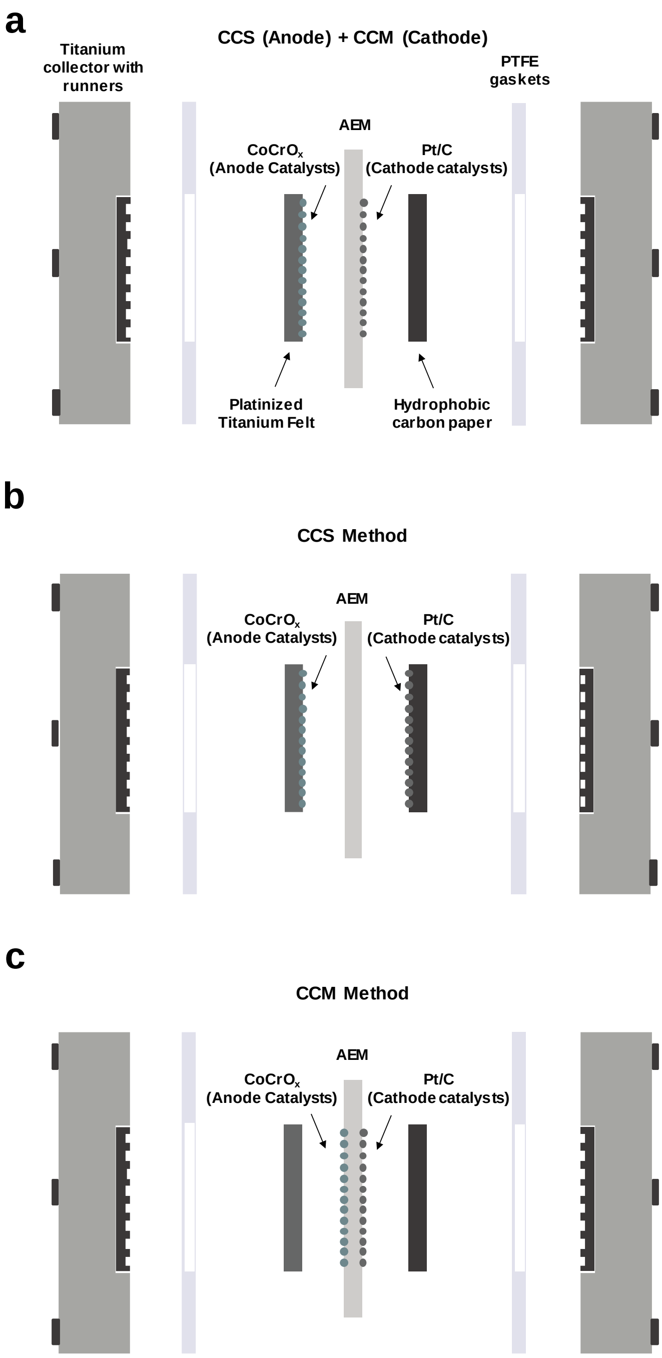

Supplementary Fig. 17. Schematic of AEMWEs using different methods. Schematic diagram of the AEMWEs with (a) anode CCS and cathode CCM method, (b) CCS method and (c) CCM method.

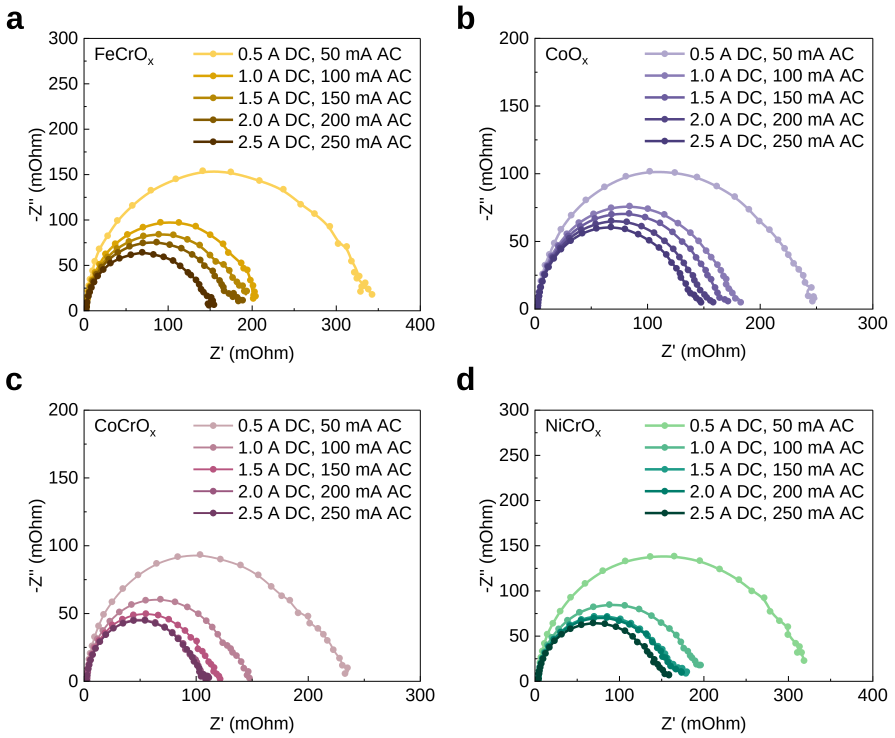

Supplementary Fig. 18. *Operando* EIS measurements. Nyquist plots of AEMWEs obtained at different currents for (a) $FeCrO_x$, (b) CoO_x, (c) $CoCrO_x$ and (d) $NiCrO_x$ as the anodes (MEA area: 5 cm^2).

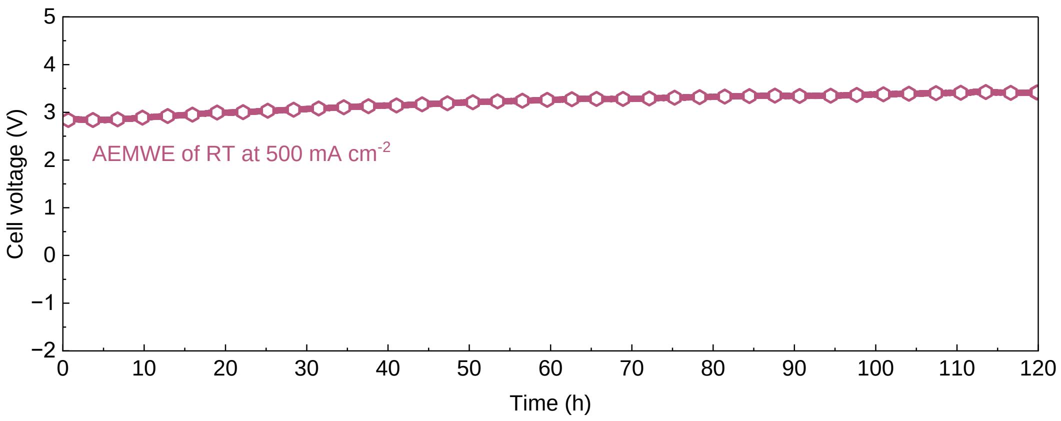

Supplementary Fig. 19. Chronopotentiometry curve. Chronopotentiometry curve of the $CoCrO_x$ catalyst at a constant current density of 0.5 A cm^{-2} in the AEMWEs at room temperature (RT).

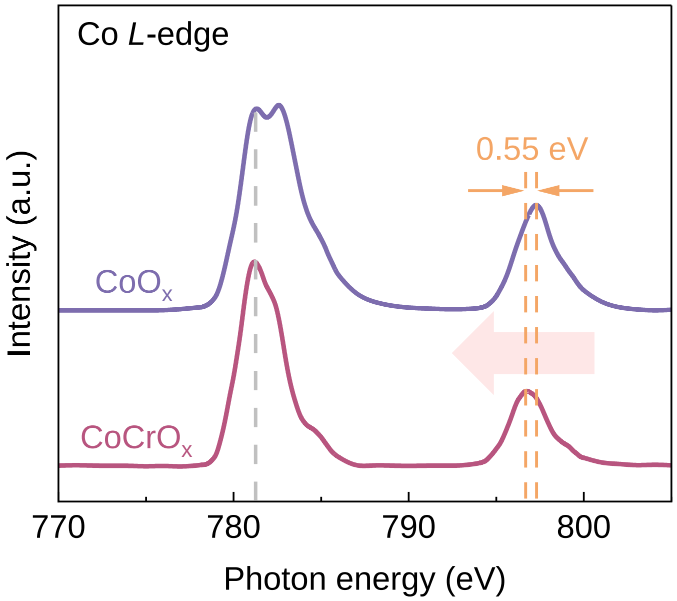

Supplementary Fig. 20. Normalized sXAS spectra. Normalized sXAS spectra of Co *L*-edge for CoO_x and $CoCrO_x$.

Note: The figure above shows that the Co L_3-edge (lower energy peak) has a higher proportion of low valence Co in $CoCrO_x$ compared to CoO_x. The left shift of $CoCrO_x$ relative to CoO_x in the L_2-edge (higher energy peak) also confirms the lower valence of Co in $CoCrO_x$.

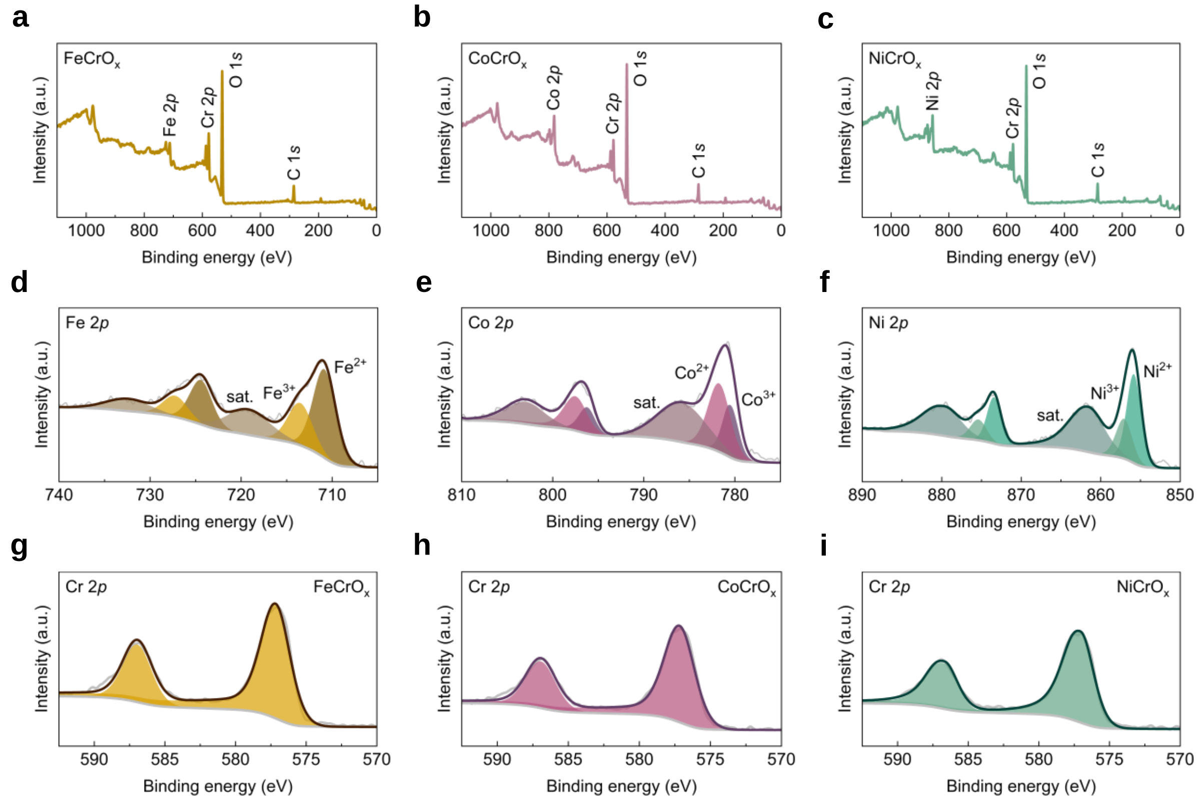

Supplementary Fig. 21. XPS spectra. XPS spectra of (a-c) survey scan for $FeCrO_x$, $CoCrO_x$ and $NiCrO_x$, (d) Fe 2*p* for $FeCrO_x$, (e) Co 2*p* for $CoCrO_x$, (f) Ni 2*p* for $NiCrO_x$, (g-i) Cr 2*p* for $FeCrO_x$, $CoCrO_x$ and $NiCrO_x$.

Note: The survey scan reveals that all catalysts contain the elements M (Fe, Co, or Ni), Cr, and O. In the Fe 2*p* spectrum, the Fe $2p_{3/2}$ and Fe $2p_{1/2}$ peaks can be divided into Fe^{2+} $2p_{3/2}$ (710.9 eV), Fe^{2+} $2p_{1/2}$ (724.5 eV), Fe^{3+} $2p_{3/2}$ (713.6 eV), and Fe^{3+} $2p_{1/2}$ (727.3 eV)[1-3]. The Co 2*p* spectrum shows that the Co $2p_{3/2}$ and Co $2p_{1/2}$ peaks at 781.8 and 797.6 eV are assigned to Co^{2+}, and those at 780.5 and 796.3 eV are assigned to Co^{3+}[4,5]. Regarding the Ni 2*p* spectrum, two peaks of $2p_{3/2}$ (855.9 eV) and $2p_{1/2}$ (873.5 eV) are assigned to Ni^{2+}, while two peaks of $2p_{3/2}$ (857.1 eV) and $2p_{1/2}$ (875.5 eV) are assigned to Ni^{3+}[6,7]. The Cr $2p_{3/2}$ for the three catalysts ranged from 577.1 to 577.3 eV and $2p_{1/2}$ from 586.7 to 587.1 eV, which is typical for Cr^{3+}[8].

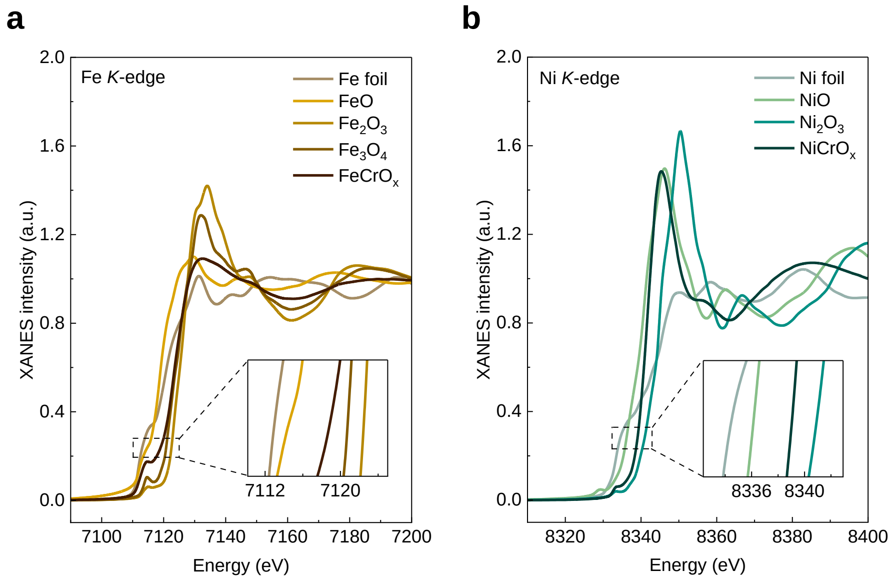

Supplementary Fig. 22. XANES spectra. XANES spectra of (a) Fe *K*-edge for $FeCrO_x$ and referenced samples and (b) Ni *K*-edge for $NiCrO_x$ and referenced samples.

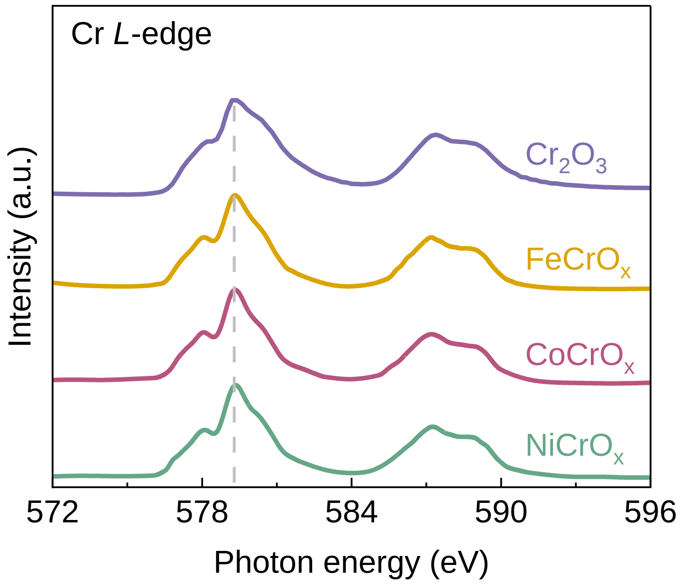

Supplementary Fig. 23. Normalized sXAS spectra. Normalized sXAS spectra of Cr *L*-edge for $FeCrO_x$, $CoCrO_x$, $NiCrO_x$ and Cr_2O_3 reference sample.

Note: The sXAS spectra of $FeCrO_x$, $CoCrO_x$, and $NiCrO_x$ exhibit comparable peak shapes and positions, which are consistent with those of the Cr_2O_3 reference sample.

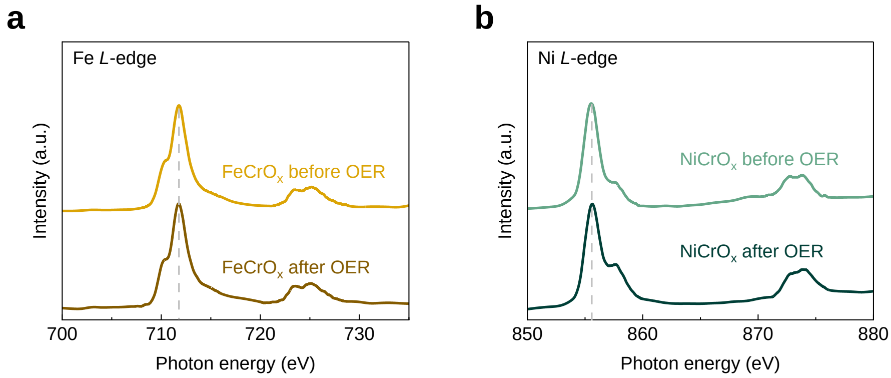

Supplementary Fig. 24. Normalized sXAS spectra before and after OER. Normalized sXAS spectra of (a) Fe *L*-edge for $FeCrO_x$, (b) Ni *L*-edge for $NiCrO_x$ before and after OER.

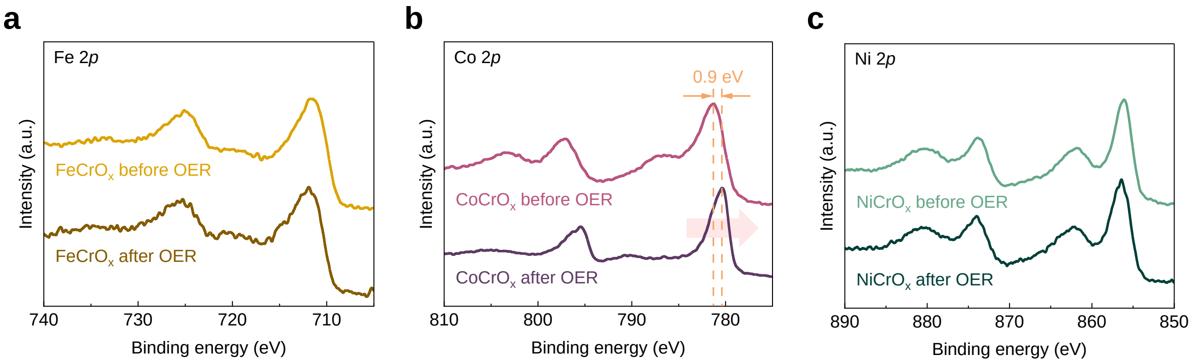

Supplementary Fig. 25. XPS spectra before and after OER. XPS spectra of (a) Fe 2*p* for $FeCrO_x$, (b) Co 2*p* for $CoCrO_x$ and (c) Ni 2*p* for $NiCrO_x$ before and after OER.

Note: As shown above, the peak shapes and positions of Fe 2*p* for $FeCrO_x$ and Ni 2*p* for $NiCrO_x$ remain almost unchanged before and after OER. However, the binding energy of Co 2*p* for $CoCrO_x$ shifts to a lower energy side by 0.9 eV.

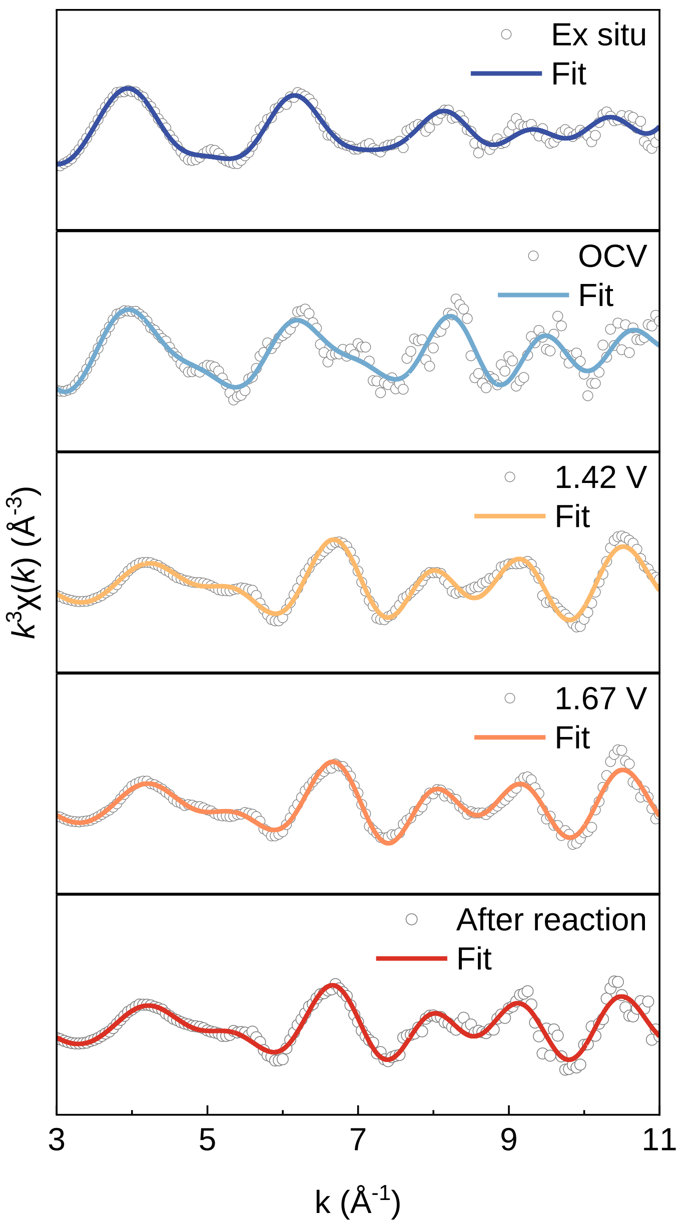

Supplementary Fig. 26. The fitting curves of $k^3\chi(k)$ oscillations. The fitting curves of $k^3\chi(k)$ oscillations of Co *K*-edge for $CoCrO_x$.

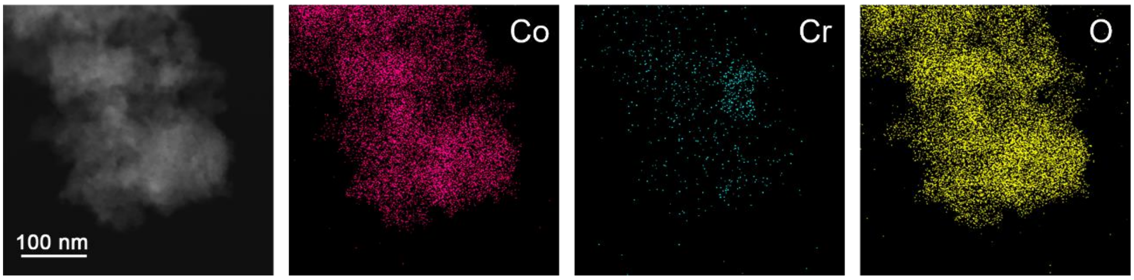

Supplementary Fig. 27. STEM image and corresponding EDS mappings. STEM image and corresponding EDS mappings of $CoCrO_x$ after the OER process showing the dispersion of Co (pink), Cr (blue-green) and O (yellow), respectively.

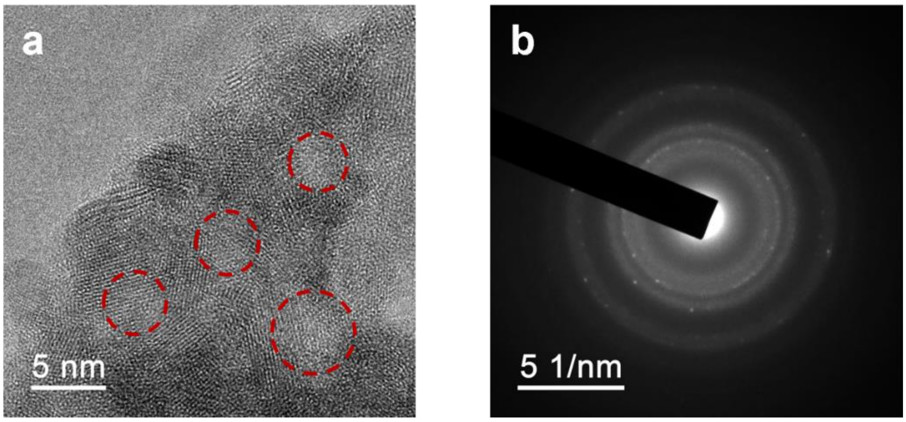

Supplementary Fig. 28. HRTEM image and SAED pattern. (a) HRTEM image (red circles indicate the new appeared nanopores) and (b) SAED pattern of $CoCrO_x$ after the OER process.

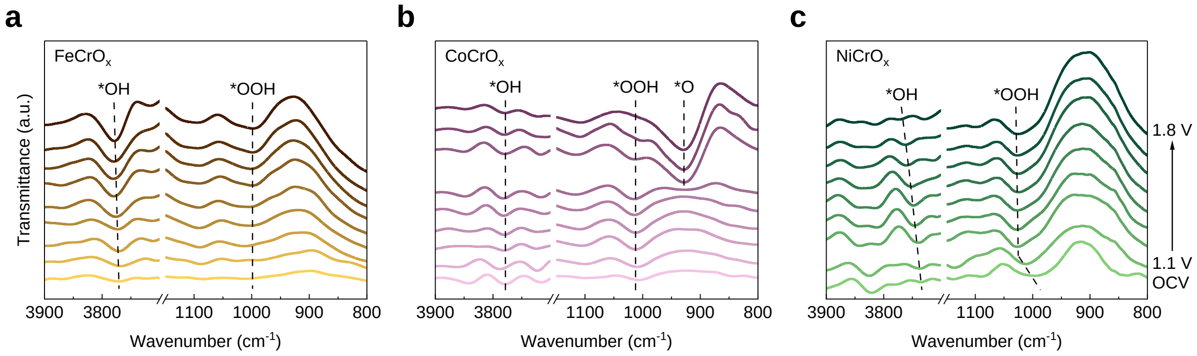

Supplementary Fig. 29. *In-situ* SR-IR measurements. *In-situ* SR-IR measurements in the range of 3900-3700 cm^{-1} and 1150-800 cm^{-1} under various potentials for (a) $FeCrO_x$, (b) $CoCrO_x$ and (c) $NiCrO_x$ during the OER process.

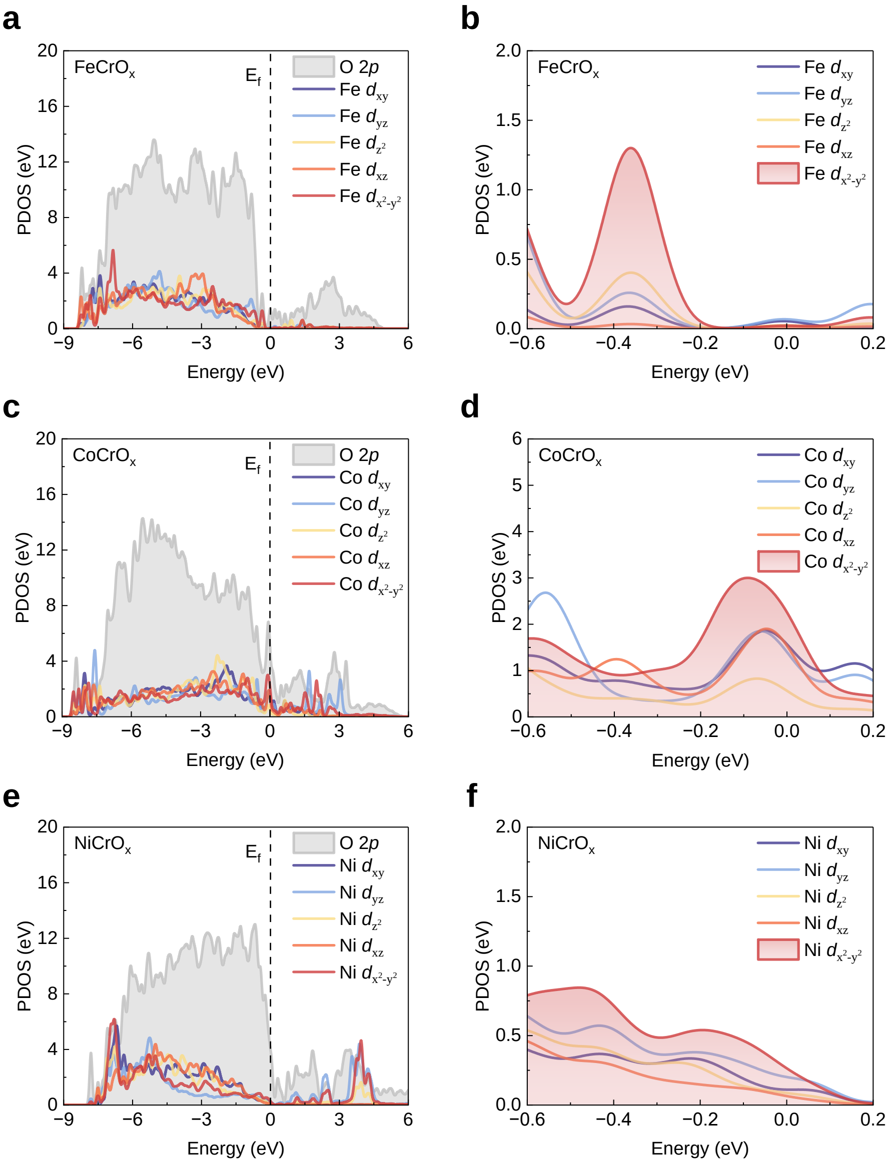

Supplementary Fig. 30. PDOS curves. PDOS of (a, b) Fe *d* and O 2*p* orbitals for $FeCrO_x$, (c, d) Co *d* and O 2*p* orbitals for $CoCrO_x$ and (e, f) Ni *d* and O 2*p* orbitals for $NiCrO_x$.

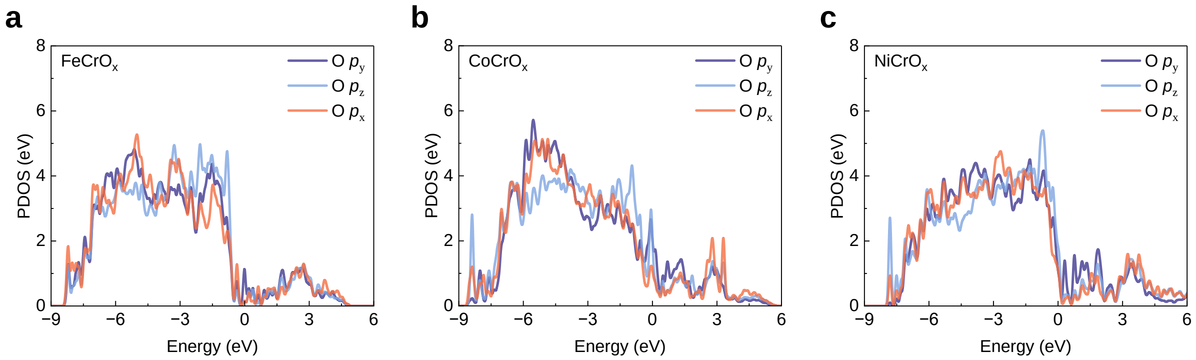

Supplementary Fig. 31. PDOS curves. PDOS of O *p* orbitals for (a) $FeCrO_x$, (b) $CoCrO_x$ and (c) $NiCrO_x$.

Supplementary Tables

Supplementary Table 1. The ICP-AES results of the $FeCrO_x$, $CoCrO_x$ and $NiCrO_x$ samples.

Content / Catalysts	Cr (*wt*%)	Fe (*wt*%)	Co (*wt*%)	Ni (*wt*%)	Atomic ratio (Cr: M)
$FeCrO_x$	25.1%	26.4%	-	-	1: 0.98
$CoCrO_x$	24.9%	-	27.1%	-	1: 0.96
$NiCrO_x$	19.9%	-	-	22.3%	1: 0.99

Supplementary Table 2. The individual electrochemical characterization data for each catalyst.

Catalysts \ Characterizations	η_{10} (mV)	η_{100} (mV)	Tafel slopes (mV dec^{-1})	C_{dl} (mF cm^{-2})	R_{ct} (Ω)	*j* in the AEM, at 2 V (A)
$FeCrO_x$	404	534	171.4	6.13	45.4	0.206
CoO_x	323	465	146.6	12.12	20.1	0.538
$CoCrO_x$	268	400	101.6	16.45	9.0	1.08
$NiCrO_x$	-	488	188.6	7.56	21.6	0.213
RuO_2	314	480	196.0	9.95	11.1	-

Note: The η_{10} data of $NiCrO_x$ is not credible due to the slight oxidation of Ni. It can be found that $CoCrO_x$ is superior in all samples.

Supplementary Table 3. The detailed XPS fitting results of Supplementary Fig. 21.

Catalyst	Element	Peak	Position (eV)	FWHM (eV)	Area
$FeCrO_x$	Fe	Fe^{2+} $2p_{3/2}$	710.9	3.0	10522.6
		Fe^{3+} $2p_{3/2}$	713.6	3.3	5442.0
		Fe $2p_{3/2}$ sat.	719.3	5.8	5747.3
		Fe^{2+} $2p_{1/2}$	724.5	3.0	5261.3
		Fe^{3+} $2p_{1/2}$	727.3	3.3	2721.0
		Fe $2p_{1/2}$ sat.	732.5	5.8	2771.2
	Cr	Cr^{3+} $2p_{3/2}$	577.3	2.5	29268.1
		Cr^{3+} $2p_{1/2}$	587.0	2.5	11556.0
$CoCrO_x$	Co	Co^{3+} $2p_{3/2}$	780.5	2.1	6538.1
		Co^{2+} $2p_{3/2}$	781.8	3.0	12074.5
		Co $2p_{3/2}$ sat.	785.9	6.5	15082.8
		Co^{3+} $2p_{1/2}$	796.3	2.1	3533.2
		Co^{2+} $2p_{1/2}$	797.6	3.0	7719.5
		Co $2p_{1/2}$ sat.	803.0	5.1	6769.3
	Cr	Cr^{3+} $2p_{3/2}$	577.2	2.6	24307.8
		Cr^{3+} $2p_{1/2}$	587.0	2.6	9625.5
$NiCrO_x$	Ni	Ni^{2+} $2p_{3/2}$	855.9	2.2	12442.1
		Ni^{3+} $2p_{3/2}$	857.1	2.5	6329.0
		Ni $2p_{3/2}$ sat.	861.7	5.0	16945.9
		Ni^{2+} $2p_{1/2}$	873.5	2.2	6317.2
		Ni^{3+} $2p_{1/2}$	875.5	2.5	3279.3
		Ni $2p_{1/2}$ sat.	879.7	5.0	11624.1
	Cr	Cr^{3+} $2p_{3/2}$	577.2	2.3	22617.6
		Cr^{3+} $2p_{1/2}$	586.8	2.3	10399.5

Supplementary Table 4. Structural parameters extracted from quantitative EXAFS curve-fitting.

Sample	Path	CN	*R*(Å)	σ^2(10^{-3}Å^2)	ΔE_0 (eV)	R-factor
Ex situ	Co-O	4.0	2.00	10.0	-4.20	0.004
	Co-Co	1.9	2.95	7.5	8.34	
	Co-Cr	3.1	3.15	9.0	0.79	
OCV	Co-O	4.1	2.01	10.0	-0.97	0.001
	Co-Co	2.1	2.80	7.5	-8.83	
	Co-Cr	3.0	3.09	9.0	-7.24	
1.42 V (*vs* RHE)	Co-O	6.1	1.90	3.8	1.17	0.017
	Co-Co	5.7	2.84	4.6	-1.24	
1.67 V (*vs* RHE)	Co-O	6.4	1.91	3.8	0.72	0.014
	Co-Co	5.4	2.83	4.6	-1.63	
After reaction	Co-O	6.0	1.90	3.8	0.34	0.004
	Co-Co	5.1	2.84	4.6	-1.07	

Note: CNs, coordination numbers; *R*, bonding distance; σ^2, Debye-Waller factor; ΔE_0, inner potential shift.

Supplementary References

1. Zhao, N. *et al.* Structure and magnetic properties of $(Fe_{1-x}Nd_x)_3N$ nanoparticles. *J. Mater. Sci.: Mater. Electron.* **29**, 13852-13857 (2018).
2. Bai, L., Wen, X. & Guan, J. High-Efficiency Electrocatalytic Water Oxidation on Trimetal-Based Fe–Co–Cr Oxide. *ACS Appl. Energy Mater.* **2**, 5584-5590 (2019).
3. Cai, W. *et al.* Amorphous versus Crystalline in Water Oxidation Catalysis: A Case Study of NiFe Alloy. *Nano Lett.* **20**, 4278-4285 (2020).
4. Wang, Z. *et al.* Cr-doped CoFe layered double hydroxides: Highly efficient and robust bifunctional electrocatalyst for the oxidation of water and urea. *Appl. Catal., B* **272**, 118959 (2020).
5. Peng, Y. *et al.* Surface Tuning of $La_{0.5}Sr_{0.5}CoO_3$ Perovskite Catalysts by Acetic Acid for NO_x Storage and Reduction. *Environ Sci Technol* **50**, 6442-6448 (2016).
6. Grosvenor, A. P., Biesinger, M. C., Smart, R. S. C. & McIntyre, N. S. New interpretations of XPS spectra of nickel metal and oxides. *Surf. Sci.* **600**, 1771-1779 (2006).
7. Zheng, X. *et al.* In Situ Fabrication of Heterostructure on Nickel Foam with Tuned Composition for Enhancing Water-Splitting Performance. *Small* **14**, e1803666 (2018).
8. Ikemoto, I. *et al.* X-ray photoelectron spectroscopic studies of CrO_2 and some related chromium compounds. *J. Solid State Chem.* **17**, 425-430 (1976).
